# Cognitive Appraisals Mediate Affective Reactivity in Affiliative Extraversion

**DOI:** 10.3389/fpsyg.2018.00782

**Published:** 2018-05-23

**Authors:** Greig Inglis, Marc C. Obonsawin, Simon C. Hunter

**Affiliations:** ^1^Scottish Collaboration for Public Health Research and Policy, University of Edinburgh, Edinburgh, United Kingdom; ^2^School of Psychological Sciences & Health, University of Strathclyde, Glasgow, United Kingdom

**Keywords:** agentic extraversion, affiliative extraversion, affective reactivity, appraisals, emotion

## Abstract

Extraversion is comprised of two main components of affiliation and agency. Affiliative and agentic extraversion have been found to predict positive activation in response to appetitive stimuli, and affiliative extraversion also predicts warmth-affection in response to affiliative stimuli. The aim of this study was to test whether cognitive appraisals could account for these personality-emotion relationships. In an online experiment, 192 participants completed affiliative and appetitive imagery tasks, and reported their affect before and after each task. Participants also reported on how they appraised the imagined events. Affiliative extraversion was positively associated with warmth-affection following the affiliative imagery, and this relationship was mediated by appraisals of intrinsic pleasantness and compatibility with internal standards. Affiliative extraversion also predicted positive activation following the affiliative imagery, and this relationship was mediated by appraisals of importance. Neither agentic nor affiliative extraversion predicted any other form of affect following either the affiliative or appetitive imagery tasks. These results suggest that cognitive appraisals may be one mechanism that mediate affective reactivity in affiliative extraversion, although future confirmatory studies are required to further test this hypothesis.

## Introduction

Extraversion consists of two main components of affiliation and agency: affiliative extraversion reflects being warm, affectionate, and valuing close relationships, and agentic extraversion reflects social dominance, assertiveness and enjoyment of leadership roles (Depue and Collins, 1999; Depue and Morrone-Strupinsky, 2005). The affiliative and agentic components are readily identifiable in several personality measures as subscales of extraversion, such as Warmth and Assertiveness in the NEO-PI-R (Costa and McCrae, 1995); Social Closeness and Social Potency in the Multidimensional Personality Questionnaire (MPQ; Tellegen and Waller, 2008); and Enthusiasm and Assertiveness in the Big Five Aspect Scales (BFAS; DeYoung et al., 2007).

Depue and colleagues have argued that agentic and affiliative extraversion reflect two emotional-motivation systems that direct behavior toward particular classes of stimuli, and that individual differences in these traits reflect variation in sensitivity to those stimuli (Depue and Collins, 1999; Depue and Morrone-Strupinsky, 2005). Specifically, agentic extraversion reflects a behavioral approach system that is sensitive to signals of reward and regulates incentive motivation and goal-directed behavior in the pursuit of those reward (Depue and Collins, 1999). The activation of this system, and the accompanying incentive motivation, is experienced as an affective state of both pleasure and high arousal (e.g., excited, enthusiastic, determined) that is known as positive activation (Watson et al., 1999). Affiliative extraversion on the other hand reflects sensitivity of a motivational system that regulates interpersonal behavior in response to affiliative stimuli, and that subsequently generates feelings of warmth and affection (Depue and Morrone-Strupinsky, 2005).

Studies of affective-reactivity in agentic and affiliative reactivity provide some support for this view. For example, agentic extraversion has been found to predict positive activation in response to appetitive stimuli (Morrone et al., 2000; Morrone-Strupinsky and Depue, 2004; Smillie et al., 2013), while affiliative extraversion has been reported to predict warmth-affection in response to affiliative stimuli (Morrone-Strupinsky and Depue, 2004; Depue and Morrone-Strupinsky, 2005). There are some inconsistencies in this literature however, as some studies do not show a relationship between affiliative extraversion and warmth-affection in response to affiliative stimuli (Morrone-Strupinsky and Lane, 2007). Moreover, some researchers have found affiliative extraversion to also predict positive activation in response to appetitive stimuli (Smillie et al., 2013), although the majority of published research shows no such relationship (Morrone et al., 2000; Morrone-Strupinsky and Depue, 2004; Morrone-Strupinsky and Lane, 2007).

Whilst previous researchers have suggested how psychological processes – such as the formation of affiliative memories (Depue and Morrone-Strupinsky, 2005) – may contribute to affective reactivity in affiliative and agentic extraversion, there have been no prior attempts to explicitly test the cognitive mechanisms that mediate these individual differences. Cognitive appraisal models of emotion could help to delineate these mechanisms and have the potential to enhance our understanding of the psychological processes that underpin affective reactivity in affiliative and agentic extraversion.

Causal appraisal models – as opposed to constitutive appraisal models (Barrett, 2014) – hold that emotions are elicited and differentiated by individuals’ appraisals of how events relate to their wellbeing (Ellsworth and Scherer, 2003). As such, it is the outcomes of these subjective evaluations that determine an individual’s emotional response, rather than the objective features of the event (Ellsworth and Scherer, 2003). Commonly suggested appraisal dimensions include intrinsic pleasantness (how pleasant the event is, regardless of the individual’s current state); goal conduciveness (the extent to which the event helps the individual meet their goals or needs) and fairness (the extent to which the outcomes of the event are considered to be fair; Ellsworth and Scherer, 2003). These appraisals glean at least four types of information: whether the event is relevant to the individual or his or her reference group (relevance); the consequences of the event and how these impact on the individual’s goals and well-being (implications); whether the individual can cope with these consequences (coping potential); and how the events relate to the individual’s self-concept and social norms (normative significance; Sander et al., 2005).

Several models of appraisal have been developed, whereby specific emotions are posited to be associated with particular patterns of appraisal (Ellsworth and Smith, 1988; Frijda et al., 1989; Roseman et al., 1996). In support of appraisal models, a large body of evidence demonstrates that particular patterns of appraisal are associated both the quality and intensity of particular emotions (Ellsworth and Scherer, 2003). In one line of research for example, individuals have been asked to recall episodes where they experienced a particular emotion, and then to rate how they appraised the emotion-eliciting event (Roseman et al., 1996; Scherer, 1997; Brans and Verduyn, 2014). In support of appraisal theory, data from these studies demonstrate that situations eliciting different emotions are associated with particular patterns of cognitive appraisal. Other researchers have adopted an experimental approach, by constructing vignettes designed to manipulate appraisals (Tracy and Robins, 2006, 2007; van Tilburg et al., 2018). For example Smith and Lazarus (1993) randomly assigned participants to complete guided imagery vignettes designed to manipulate several appraisal dimensions, before asking participants to rate how they would feel in those situations. The results demonstrated that participants’ ratings of anger, guilt and fear/anxiety differed across the conditions, in patterns consistent with appraisal theories. Further evidence still shows that appraisals predict the intensity of recently occurring emotional experiences. Siemer et al. (2007) for example found that appraisals predicted participants’ ratings of six emotions – including anger, guilt and shame – in response to a stressful laboratory task. Tong et al. (2007) report similar findings from an experience sampling study, where appraisals were found to predict the reported intensity of six naturally occurring emotions, including anger, sadness and fear.

These studies are typical of the majority of appraisal research, in that they focus on specific, discrete affects. Appraisal theories are flexible in the number of appraisals that are processed in a given situation however, and several theorists predict that the emotional response to an event will be relatively broad and undifferentiated when only a few appraisals are made (Moors et al., 2013). Appraisals may therefore also be applicable to broad affective dimensions highlighted in dimensional models of affect, such as valance and activation (Russell and Barrett, 1999) or positive and negative activation (Watson et al., 1999). In support of this view, appraisals have been found to predict ratings of both valance and arousal in response to viewing pictures (Scherer et al., 2006) and in response to daily events (Kuppens et al., 2012).

If cognitive appraisals can at least partly account for the differentiation and intensity of emotions, then appraisals may be one mechanism underlying affective reactivity in agentic and affiliative extraversion. Indeed, a key feature of appraisal theory is the ability of this approach to account for individual differences in emotional responses to the same situation (Moors et al., 2013). Specifically, appraisal researchers have suggested that individual differences in emotional experiences can be attributed to stable differences in how individuals appraise particular situations (Kuppens and Tong, 2010). For example, vulnerability to depression may be explained by a tendency to appraise events in a manner that generates more frequent or intense experiences of sadness or despair in daily life (Mehu and Scherer, 2015). Although there is no evidence of how affiliative or agentic extraversion specifically may be related to specific appraisals, there is evidence that other personality traits are associated with appraisals, which broadly support the view that appraisals can account for personality differences in affective experience. For example, neuroticism is associated with the tendency to appraise events as being unfair, obstructive to goals, uncontrollable, uncertain and as violating moral standards (Tong et al., 2006). Stressor-related appraisals have also been found to partially mediate the relationships between neuroticism, conscientiousness and openness and stressor-related negative affect (Leger et al., 2016).

The aims of this study were to test affective reactivity in agentic and affiliative extraversion, and to explore the possibility that cognitive appraisals could account for these individual differences in affective reactivity. It was predicted that affiliative extraversion would predict warmth-affection in response to an affiliative stimulus and that agentic extraversion would predict positive activation following an agentic stimulus. No predictions were made concerning the ways in which affiliative extraversion might be associated with affect following an appetitive stimulus due to the inconsistent data to date. Although some research supports a relationship between affiliative extraversion and positive activation following an appetitive stimulus (Smillie et al., 2013), or suggests that these constructs may be relatively weakly related (Morrone et al., 2000), other research does not (Morrone-Strupinsky and Depue, 2004; Morrone-Strupinsky and Lane, 2007).

We also expected that the relationships between agentic and affiliative extraversion and affect would be mediated by cognitive appraisals. It was difficult to make confident predictions about which appraisals would mediate these relationships however, as there is currently a lack of research on whether specific appraisals are associated with either agentic or affiliative extraversion. Moreover, positive effects are often relatively undifferentiated in appraisal models of emotion and so there is little data on the appraisal dimensions that predict either positive activation or warmth-affection. Ellsworth and Smith (1988) however report that feelings of “hope/confidence” (*hopeful, expectant, confident, proud* and *triumphant*) are associated with appraisals of pleasantness, self-agency, effort, predictability and importance. Therefore, we tentatively predicted that these appraisals would mediate the relationships between agentic extraversion and positive activation. These authors further report that feelings of “love” (*loving, friendly, admiring, grateful*) are positively associated with appraisals of pleasantness, other agency, importance and negatively associated with appraisals of effort. Therefore, we tentatively predicted that these appraisals would mediate the relationship between affiliative extraversion and warmth-affection.

## Materials and Methods

### Participants

A total of 192 participants (132 females) took part in the experiment online, with a mean age of 26.33 years (*SD* = 11.86). The most common nationalities listed by participants were American (57.81%) followed by British (21.05%). The sample size was informed by rules of thumb for determining the number of cases necessary for regression analyses. First, a sample size of 107 participants would be required to detect a medium effect size between affiliative or agentic extraversion and affect in a model with three predictor variables (Tabachnick and Fidell, 2007). Secondly, a sample size of 148 participants would be necessary to detect a mediation effect where the effect sizes between the independent, dependent and mediating variables are approximately halfway between small and medium effects (Fritz and MacKinnon, 2007).

### Emotion Induction Vignettes

Participants were presented with two vignettes. The vignette to induce warmth-affection reflected the content of the film developed by Morrone-Strupinsky and Depue (2004). Participants were asked to imagine themselves participating in an affectionate exchange with a romantic partner and newborn child. The other vignette was designed to induce positive activation, whereby participants were asked to imagine themselves buying a lottery ticket and winning £1000. This vignette has previously been demonstrated to be effective in inducing positive activation in studies of affective reactivity (Smillie et al., 2012).

### Measures

Affiliative and agentic extraversion were measured with the Enthusiasm (*α* = 0.84) and Assertiveness (*α* = 0.88) scales of the Big Five Aspect Scales, respectively (BFAS; DeYoung et al., 2007). Each scale is made up of ten items each, and each item is rated on a five point Likert scale. The Enthusiasm scale consists of items such as “I warm up easily to others” and “I am hard to get to know” (reverse scored). The Assertiveness scale consists of items such as “I take charge” and “I have a strong personality.” In the current sample, Enthusiasm and Assertiveness were moderately correlated (*r* = 0.46), which is consistent with previous research (DeYoung et al., 2007). The BFAS scales are highly correlated with other measures of agentic and affiliative components of extraversion, such as MPQ Social Closeness and Social Potency (DeYoung et al., 2013), and with the overall domain of extraversion (DeYoung et al., 2007). Moreover, these scales have been used as measures of affiliative and agentic extraversion in previous studies of affective reactivity (Smillie et al., 2013).

Given the lack of previous research on how the agentic and affiliative components of extraversion might relate to appraisals, we sought to sample a comprehensive set of appraisal dimensions. As there is no consensus on how many appraisals are sufficient to account for emotional experiences, the works of several theorists’ were reviewed to identify common dimensions (Ellsworth and Smith, 1988; Frijda et al., 1989; Roseman et al., 1996; Lazarus, 2001; Scherer, 2001). Twelve appraisal dimensions were identified, each of which was measured with between 1–3 items. The items were adapted from measures previously developed by the researchers noted above, and the phrasing of these were altered slightly to reflect either the family or lottery conditions. Participants rated each item on a scale from 1 (not at all) to 5 (completely). Reliabilities and examples of the items included in each measure are displayed in **Table 1**. The full scales are provided in Supplementary Material 1.

**Table 1:** Reliabilities and example items of the appraisal scales.

Appraisal	No. of items	Example item	*α* family condition	*α* lottery condition
Intrinsic pleasantness	1	How pleasant would this family interaction be in general, regardless of your current needs, desires or feelings?		
Importance	3	How important was this family interaction to you?	0.70	0.80
Situational agency	3	To what extent did this family interaction occur by chance?	0.82	0.54
Self-agency	3	How responsible were you for this family interaction occurring?	0.85	0.64
Other-agency	3	How responsible was another person for what happened in this family interaction?	0.92	0.93
Outcome probability	3	To what extent did you think that the outcome of this family interaction clearly predictable?	0.84	0.90
Goal conduciveness	3	To what extent did you think that this family interaction would have positive consequences for you?	0.88	0.74
Controllability	2	To what extent could a person (either you or another person) influence the outcome of this family interaction?	0.76	0.61
Power	2	To what extent did you think that you were able to control the potential consequences of this family interaction?	0.88	0.78
Compatibility with internal standards	3	To what extent was this family interaction consistent with your personal beliefs, values and ideals?	0.90	0.84
Effort	3	How much effort (mental or physical) did you feel you had to expend during this family interaction?	0.82	0.75
Fairness	3	To what extent did you think that what happened to you in this family interaction was fair?	0.83	0.82

We assessed this new multi-dimensional model of appraisals using a confirmatory factor analysis. The CFA was conducted using AMOS22.0, and our model involved 11 latent variables (Importance, Situational-Agency, Self-Agency, Other-Agency, Outcome Probability, Goal Conduciveness, Controllability, Power, Compatibility with Internal Standards, Effort and Fairness), each of which had items loading as per the descriptions in **Table 1**. All latent variables were allowed to co-vary. In addition, the single-item used to assess Intrinsic Pleasantness was included in the model by allowing it to co-vary with each of the 11 latent variables.

The fit of the model was acceptable for the ‘lottery’ condition: CMIN/DF = 1.72, CFI = 0.920, RMSEA = 0.061 (90%CI = 0.053,0.069), SRMR = 0.074. Fit was also acceptable for the ‘family’ condition: CMIN/DF = 1.61, CFI = 0.937, RMSEA = 0.057 (90%CI = 0.048,0.065), SRMR = 0.058. Taken together, these results indicate that the measurement model for appraisals was appropriate.

Warmth-affection was measured with Diener et al. (1995) four item “Love” scale (*love, caring, fondness* and *affection)*. Positive activation was measured with the Positive Activation scale of the PANAS (Watson et al., 1988). Participants rated each item of both affect measures on a five-point scale to indicate the extent to which they feel “right now, that is at the present moment.” These measures were completed before each imagery task to assess baseline affect, and after each task to assess participants’ affective responses. Cronbach *α* for these scales ranged between 0.92 and 0.96.

### Procedure

The study was approved by the Ethics Committee of School of Psychological Sciences and Health at University of Strathclyde, and all participants provided written informed consent. The study was conducted online, and was advertised on social media and websites dedicated to recruiting participants for online psychology experiments.

The experiment was displayed through Qualtrics^1^. Participants first completed the BFAS scales, followed by the affect questionnaires. Participants were then presented with one of the vignettes and were instructed to imagine themselves experiencing the situation as vividly as possible. These instructions remained onscreen for 90 s, after which participants completed the affect questionnaires for a second time, and then completed the appraisal questionnaire to rate how they had appraised the previously imagined situation. Participants then completed the affect questionnaires again in order to record a new baseline measure, before the process was repeated with the second imagery vignette. The order in which each imagery condition was presented was counterbalanced between participants.

## Results

The first set of analyses tested whether the lottery and family vignettes induced states of positive activation and warmth-affection. Descriptive statistics are presented in **Table 2**.

**Table 2:** Means (SD) of affect scores pre and post the family and lottery conditions.

	Affiliative family imagery		Appetitive lottery imagery	
	Pre	Post	Pre	Post
Positive activation	28.18 (9.22)	37.47 (9.68)*	28.88 (10.14)	36.91 (10.34)*
Warmth-affection	11.45 (4.60)	17.69 (3.85)*	12.08 (4.79)	12.50 (4.94)

*^∗^Pre–post difference in affect score significant at *p* < 0.001.*

Several affect scores were not normally distributed. Square root transformations improved the distribution of most variables, and these transformed data were used in the subsequent manipulation checks. Transformations did not improve the distribution of warmth-affection scores following the affiliative imagery however, and so these data were analyzed with a Wilcoxon signed-rank test.

Following the affiliative imagery, there were large increases in warmth-affection, *z* = -10.88, *p* < 0.001, *r* = 0.55 and positive activation *t*(191) = -12.18, *p* < 0.001, *r* = 0.66. Positive activation scores also increased following the appetitive imagery, *t*(191) = -11.40, *p* < 0.001, *r* = 0.64, although there was no change in warmth-affection scores, *t*(191) = -1.24, *p* = 0.217, *r* = 0.09.

### Testing the Relationships Between Personality, Affect and Appraisals Following the Affiliative Family Imagery

The next set of analyses tested whether personality traits predicted affect following the affiliative imagery, and whether these relationships were mediated by appraisals. Descriptive statistics are reported in **Table 3**. As some data were heavily skewed, non-parametric Spearman’s correlations were conducted.

**Table 3:** Descriptive statistics and correlations between personality, affect and appraisals in the family imagery condition.

	2	3	4	5	6	7	8	9	10	11	12	13	14	15	16	17	18	Mean (*SD*)
(1) Enthusiasm	0.46^∗∗∗^	0.45^∗∗∗^	0.31^∗∗∗^	0.39^∗∗∗^	0.35^∗∗∗^	0.21^∗∗^	0.25^∗∗^	0.18^∗^	0.06	0.09	0.15^∗^	0.23^∗∗^	0.13	0.15^∗^	0.26^∗∗∗^	-0.08	0.13	3.58 (0.69)
(2) Assertiveness		0.32^∗∗∗^	0.15^∗^	0.39^∗∗∗^	0.33^∗∗∗^	0.09	0.09	0.14	0.02	0.05	0.15^∗^	0.08	0.15^∗^	0.14^∗^	0.09	0.02	0.07	3.32 (0.76)
(3) Pre warmth-affection			0.29^∗∗∗^	0.71^∗∗∗^	0.45^∗∗∗^	0.07	0.26^∗∗∗^	0.17^∗^	0.02	0.06	0.12	0.16^∗^	0.10	0.22^∗∗^	0.22^∗∗^	0.12	0.16^∗^	11.45 (4.60)
(4) Post warmth-affection				0.22^∗∗^	0.70^∗∗∗^	0.63^∗∗∗^	0.59^∗∗∗^	0.32^∗∗∗^	0.01	0.20^∗^	0.04	0.49^∗∗∗^	0.19^∗∗^	0.33^∗∗∗^	0.60^∗∗∗^	-0.14	0.43^∗∗∗^	17.69 (3.85)
(5) Pre positive activation					0.43^∗∗∗^	0.08	0.21^∗∗^	0.23	0.06	0.10	0.11	0.11	0.19^∗∗^	0.30^∗∗∗^	0.25^∗∗^	0.10	0.18^∗^	28.18 (9.22)
(6) Post positive activation						0.44^∗∗∗^	0.63^∗∗∗^	0.35^∗∗∗^	0.03	0.19^∗∗^	0.15^∗^	0.46^∗∗∗^	0.20^∗∗^	0.30^∗∗∗^	0.53^∗∗∗^	0.07	0.43^∗∗∗^	37.47 (9.68)
(7) Intrinsic pleasantness							0.62^∗∗∗^	0.28^∗∗∗^	-0.05	0.20^∗∗^	0.04	0.51^∗∗∗^	0.18^∗^	0.27^∗∗∗^	0.54^∗∗∗^	-0.30^∗∗∗^	0.38^∗∗∗^	4.55 (0.76)
(8) Importance								0.39^∗∗∗^	-0.03	0.29^∗∗∗^	0.05	0.68^∗∗∗^	0.31^∗∗∗^	0.34^∗∗∗^	0.67^∗∗∗^	-0.08	0.46^∗∗∗^	12.77 (2.26)
(9) Self-agency									-0.01	0.49^∗∗∗^	0.11	0.43^∗∗∗^	0.41^∗∗∗^	0.55^∗∗∗^	0.40^∗∗∗^	0.10	0.46^∗∗∗^	11.01 (2.63)
(10) Situational-agency										0.00	0.10	-0.09	-0.01	0.02	-0.11	0.32^∗∗∗^	-0.10	7.83 (3.24)
(11) Other-agency											0.07	0.37^∗∗∗^	0.61^∗∗∗^	0.48^∗∗∗^	0.28^∗∗∗^	0.02	0.36^∗∗∗^	11.19 (2.75)
(12) Outcome probability												0.19^∗∗^	0.19^∗∗^	0.18^∗^	0.16^∗^	0.15^∗^	0.15^∗^	9.81 (2.79)
(13) Goal conduciveness													0.41^∗∗∗^	0.44^∗∗∗^	0.72^∗∗∗^	-0.12	0.53^∗∗∗^	12.44 (2.61)
(14) Controllability														0.49^∗∗∗^	0.36^∗∗∗^	0.04	0.36^∗∗∗^	7.64 (1.77)
(15) Power															0.46^∗∗∗^	0.13	0.50^∗∗∗^	7.58 (1.77)
(16) Compatibility with internal standards																-0.19^∗∗^	0.59^∗∗∗^	12.37 (2.73)
(17) Effort																	0.01	7.84 (3.14)
(18) Fairness																		12.10 (2.47)

*^∗^*p* < 0.05, ^∗∗^*p* ≤ 0.01, ^∗∗∗^*p* < 0.001.*

Two hierarchical multiple regressions were conducted to test whether Enthusiasm or Assertiveness predicted affect following the affiliative imagery task. In the first model, post-imagery warmth-affection scores were regressed on baseline warmth-affection scores in the first step, followed by Enthusiasm and Assertiveness in the second step. In the second model, post-imagery positive activation scores were regressed on baseline positive activation scores in the first step, followed by Enthusiasm and Assertiveness scores in the second.

Enthusiasm predicted both post-imagery warmth-affection (*β* = 0.23, *p* = 0.006) and positive activation (*β* = 0.17, *p* = 0.027), whilst Assertiveness did not predict either warmth-affection *β* = -0.02, *p* = 0.851) or positive activation (*β* = 0.13, *p* = 0.083). Further regression analyses were then conducted in order to identify which appraisals were predicted by Enthusiasm, and should therefore be included in the subsequent mediation analyses. Assertiveness was included as a covariate in each analysis and as these analyses were exploratory, no corrections were made for multiple tests (Streiner and Norman, 2011). After controlling for Assertiveness, Enthusiasm was found to predict pleasantness (*β* = 0.22, *p* = 0.007), importance (*β* = 0.26, *p* = 0.001), goal conduciveness (*β* = 0.24, *p* = 0.003) and compatibility with internal standards (*β* = 0.28, *p* < 0.001).

**FIGURE 1:**
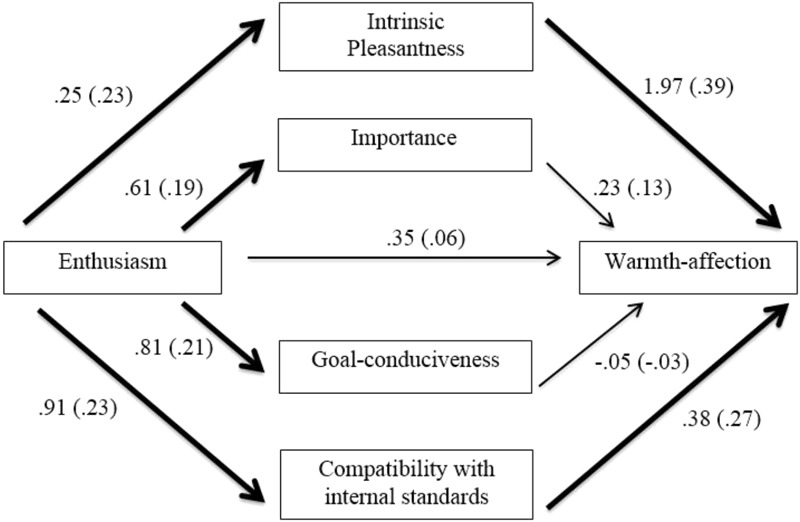
Mediation model of affiliative extraversion and warmth-affection through appraisals. All coefficients represent unstandardized (standardized) regression coefficients, controlling for Assertiveness and baseline warmth-affection scores. Bold lines represent significant coefficients, *p* < 0.05.

The PROCESS macro provided by Hayes (2013) was used to test whether the four identified appraisals mediated the relationships between Enthusiasm and warmth-affection or positive activation. The first analysis tested whether these appraisals mediated the relationship between Enthusiasm and warmth-affection following the family imagery, while controlling for baseline warmth-affection and Assertiveness. The results of are presented in **Figure 1** and **Table 4**.

**Table 4:** Unstandardized (B) and standardized (β) coefficients, standard errors and bias-corrected confidence intervals of the mediation model testing the indirect effects of the affiliative extraversion on warmth-affection through appraisals following the family imagery.

				*B* 95% BC CI	
	*B*	*β*	*B* SE	Lower	Upper
Total	0.95	0.17	0.47	0.0578	1.9340
Intrinsic pleasantness	0.50	0.09	0.31	0.0049	1.2409
Importance	0.14	0.03	0.15	-0.0357	0.6214
Goal conduciveness	-0.04	-0.01	-0.38	-0.3786	0.1424
Compatibility with internal standards	0.35	0.06	0.20	0.0608	0.9086

*BC CI, Bias-corrected confidence intervals based on 10,000 bootstrapped samples.*

**FIGURE 2:**
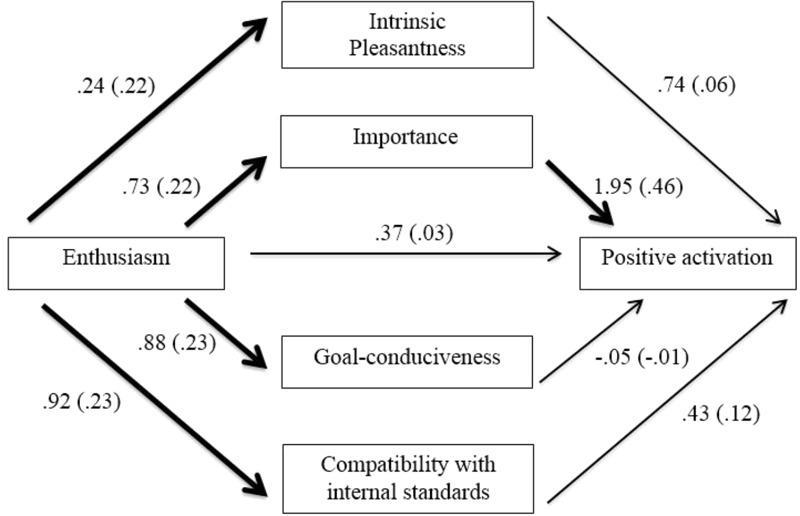
Mediation model of affiliative extraversion and positive activation through appraisals. All coefficients represent unstandardized (standardized) regression coefficients, controlling for Assertiveness and baseline positive activation scores. Bold lines represent significant coefficients, *p* < 0.05.

The indirect effects of intrinsic pleasantness and compatibility with internal standards were significant, and these appraisals therefore mediated the relationship between Enthusiasm and warmth-affection. The completely standardized indirect effect of intrinsic pleasantness was 0.09, meaning that a one standard deviation increase in Enthusiasm was associated with 0.09 of a standard deviation increase in warmth-affection, due to the relationship between Enthusiasm and appraisals of intrinsic pleasantness, which in turn predicted warmth-affection. The completely standardized indirect effect of compatibility with internal standards was 0.06, meaning that a one standard deviation increase in Enthusiasm was associated with 0.06 of a standard deviation increase in warmth-affection, due to the association between Enthusiasm and appraisals of compatibility with internal standards, which in turn predicted warmth-affection. The next analysis tested whether appraisals mediated the relationship between Enthusiasm and positive activation. The results are presented in **Figure 2** and **Table 5**.

**Table 5:** Unstandardized (B) and standardized (β), standard errors and bias-corrected confidence intervals of the mediation model testing the indirect effects of the affiliative extraversion on positive activation through appraisals following the family imagery.

				*B* 95% BC CI	
	*B*	*β*	*B* SE	Lower	Upper
Total	1.96	0.14	0.96	0.1168	3.8373
Intrinsic pleasantness	0.18	0.01	0.27	-0.1868	0.9643
Importance	1.42	0.10	0.79	0.0269	3.2265
Goal conduciveness	-0.04	0.00	0.30	-0.7408	0.5363
Compatibility with internal standards	0.40	0.03	0.34	-0.0459	1.4154

*BC CI, Bias-corrected confidence intervals based on 10000 bootstrapped samples.*

The indirect effect of importance was significant, and this appraisal therefore mediated the relationship between Enthusiasm and positive activation. The completely standardized indirect effect was 0.10, meaning that a one standard deviation increase in Enthusiasm was associated with a 0.10 standard deviation increase in positive activation, due to the relationship between enthusiasm and appraisals of importance, which in turn predicted positive-activation.

### Testing the Relationships Between Personality, Affect and Appraisals Following the Appetitive Lottery Imagery

The next set of analyses tested whether affiliative or agentic extraversion predicted affect following the lottery imagery. The descriptive statistics are presented in **Table 6**. As some data were heavily skewed, non-parametric Spearman’s correlations are reported.

**Table 6:** Descriptive statistics and correlations between personality, affect and appraisals in the lottery imagery condition.

	2	3	4	5	6	7	8	9	10	11	12	13	14	15	16	17	18	Mean (*SD*)
(1) Enthusiasm	0.46^∗∗∗^	0.48^∗∗∗^	0.33^∗∗∗^	0.42^∗∗∗^	0.35^∗∗∗^	0.11	0.03	0.21^∗∗^	0.06	0.08	0.17^∗^	0.05	0.12	0.17^∗^	0.16^∗^	0.00	0.16^∗^	3.58 (0.69)
(2) Assertiveness		0.33^∗∗∗^	0.24^∗∗^	0.38^∗∗∗^	0.29^∗∗∗^	0.11	0.09	0.16^∗^	-0.03	0.04	0.16^∗^	0.04	0.09	0.16^∗^	0.14^∗^	0.05	0.17^∗^	3.32 (0.76)
(3) Pre warmth-affection			0.53^∗∗∗^	0.80^∗∗∗^	0.51^∗∗∗^	0.18^∗^	0.02	0.07	0.15^∗^	0.01	0.08	0.07	0.05	0.01	0.10	-0.03	0.16^∗^	12.08 (4.79)
(4) Post warmth-affection				0.51^∗∗∗^	0.82^∗∗∗^	0.22^∗∗^	0.19^∗∗^	0.12	0.13	0.12	0.15	0.23	0.09	0.11	0.27^∗∗∗^	0.13	0.28^∗∗∗^	12.50 (4.94)
(5) Pre positive activation					0.53^∗∗∗^	0.10	0.03	0.10	0.07	0.05	0.13	0.03	0.05	0.05	0.12	0.02	0.12	28.88 (10.14)
(6) Post positive activation						0.39^∗∗∗^	0.23^∗∗∗^	0.10	0.24^∗∗^	0.01	0.04	0.39^∗∗∗^	0.08	0.07	0.24^∗∗^	0.09	0.34^∗∗∗^	36.91 (10.34)
(7) Intrinsic pleasantness							0.25^∗∗^	0.05	0.28^∗∗∗^	-0.14	-0.18^∗^	0.44^∗∗∗^	-0.02	-0.02	0.03	0.01	0.29^∗∗∗^	4.61 (0.70)
(8) Importance								0.36^∗∗∗^	0.12	0.23^∗∗^	0.27^∗∗∗^	0.60^∗∗∗^	0.27^∗∗∗^	0.25^∗∗∗^	0.33^∗∗∗^	0.44^∗∗∗^	0.49^∗∗∗^	10.43 (3.22)
(9) Self-agency									-0.14	0.63^∗∗∗^	0.67^∗∗∗^	0.16^∗^	0.54^∗∗∗^	0.63^∗∗∗^	0.53^∗∗∗^	0.51^∗∗∗^	0.41^∗∗∗^	7.12 (2.99)
(10) Situational-agency										-0.13	-0.16^∗^	0.33^∗∗∗^	-0.01	-0.10	0.03	-0.06	0.17^∗^	12.21 (2.55)
(11) Other-agency											0.71^∗∗∗^	0.00	0.56^∗∗∗^	0.62^∗∗∗^	0.42^∗∗∗^	0.51^∗∗∗^	0.22^∗∗^	5.54 (3.29)
(12) Outcome probability												-0.02	0.62^∗∗∗^	0.64^∗∗∗^	0.52^∗∗∗^	0.57^∗∗∗^	0.27^∗∗∗^	5.28 (3.21)
(13) Goal conduciveness													0.07	0.04	0.20^∗∗^	0.20^∗∗^	0.48^∗∗∗^	12.02 (2.31)
(14) Controllability														0.66^∗∗∗^	0.47^∗∗∗^	0.48^∗∗∗^	0.28^∗∗∗^	4.58 (2.27)
(15) Power															0.42^∗∗∗^	0.42^∗∗∗^	0.18^∗^	4.04 (2.41)
(16) Compatibility with internal standards																0.46^∗∗∗^	0.49^∗∗∗^	7.78 (3.14)
(17) Effort																	0.29^∗∗∗^	7.41 (3.08)
(18) Fairness																		9.84 (3.25)

*^∗^*p* < 0.05, ^∗∗^*p* ≤ 0.01, ^∗∗∗^*p* < 0.001.*

Two hierarchical multiple regressions were conducted to test whether Assertiveness or Enthusiasm predicted affect following the lottery imagery. In the first model, post-imagery positive activation scores were regressed on baseline positive activation scores in the first step, followed by Assertiveness and Enthusiasm scores in the second. In the second model, post-imagery warmth-affection scores were regressed on baseline warmth-affection scores in the first step, followed by Assertiveness and Enthusiasm in the second step. Post-imagery positive activation scores were not associated with either Assertiveness (*β* = 0.06, *p* = 0.424) or Enthusiasm (*β* = 0.14, *p* = 0.063), nor were warmth-affection scores associated with either Assertiveness (*β* = 0.04, *p* = 0.554) or Enthusiasm (*β* = 0.08, *p* = 0.265).

## Discussion

The aims of this study were to test affective reactivity in affiliative and agentic extraversion, and to test whether cognitive appraisals account for these individual differences. Affiliative – but not agentic - extraversion predicted feelings of warmth-affection and positive activation following an affiliative imagery task. The relationship between affiliative extraversion and warmth-affection was mediated by appraisals of intrinsic pleasantness and compatibility with internal standards, whilst the relationship between affiliative extraversion and positive activation was mediated by appraisals of importance. Neither appetitive nor affiliative extraversion predicted positive activation in response to an appetitive imagery task.

### Examining the Relationships Between Personality and Affect

Affiliative extraversion predicted both warmth-affection and positive activation in response to an affiliative stimulus, while simultaneously controlling for baseline affect and agentic extraversion. Previous researchers have reported a similar relationship between affiliative extraversion and warmth-affection (Morrone-Strupinsky and Depue, 2004), but evidence for the relationship between affiliative extraversion and positive activation has been mixed. Smillie et al. (2013) found affiliative extraversion to predict positive activation in response to an appetitive stimulus, but other researchers have found no such association following either an appetitive or affiliative stimulus (Morrone et al., 2000; Morrone-Strupinsky and Depue, 2004; Morrone-Strupinsky and Lane, 2007).

One key difference between studies reporting an association between affiliative extraversion and positive activation and those that have not is the personality measure used. Morrone-Strupinsky and Depue (2004), for example, measured affiliative extraversion with the MPQ Social Closeness scale, whilst the current study and Smillie et al. (2013) employed the BFAS Enthusiasm scale. The BFAS combines items that assess affiliation (e.g., “warm up quickly to others”) and positive affectivity (e.g., “have a lot of fun”; DeYoung et al., 2007), whereas MPQ Social Closeness does not contain items that relate to positive affectivity. Measures of affiliative extraversion that include items pertaining to positive affectivity may be more strongly related to positive activation than those that do not. In future research it would therefore be advantageous to include multiple measures of affiliative extraversion to test how these scales differ in their predictive power.

Contrary to our predictions, agentic extraversion did not predict positive activation in response to an appetitive stimulus. This may in part be due to the content of the stimulus that we employed, and the instruction to participants to imagine themselves winning a lottery. Previous investigators have demonstrated that this vignette is effective in inducing positive activation, and that the intensity of positive activation reported by participants is associated with trait extraversion (Smillie et al., 2012). Our results demonstrate that the intensity of positive activation after this vignette is not associated with agentic extraversion, that that may be because the vignette does not emphasize the goal-striving and approach behaviors that are characteristic of agentic extraversion. Winning a lottery may be more associated with perceptions of luck than with self-agency. This interpretation is consistent with the fact that while Assertiveness correlated with self-agency appraisals – but not situational or other-agency – only appraisals of situational agency were associated with positive activation following the appetitive scenario. Appetitive stimuli that describe goal-directed behavior leading to goal attainment in greater detail may produce states of positive activation that are more reliably associated with agentic extraversion (Smillie et al., 2013).

### Appraisals as Mediators of the Relationships Between Personality and Affect

The results of this study support the view that appraisals can account for at least some individual differences in affective experience (Kuppens and Tong, 2010), and therefore indicate a potential psychological mechanism underlying personality differences in responsiveness to affiliative stimuli. These findings also resonate with social-cognitive accounts of personality, such as the Cognitive-Affective Processing System (CAPS) model. According to the CAPS model, individuals’ personality consists of a complex network of cognitive-affective units that include mental representations of situations, the self and others; expectations and beliefs; motives, goals and values; and self-regulation skills. According to this model, individuals’ behavioral, cognitive and affective responses to situations are largely determined by the content and organization of these various cognitive-affective units (Mischel and Shoda, 2008). These cognitive-affective units also influence appraisals (Shoda and Smith, 2004), and so the appraisals identified in this study may also provide some insight into the social-cognitive elements that are central to trait affiliative extraversion.

It was predicted that individual differences in warmth-affection would be mediated by appraisals of intrinsic pleasantness, other agency, importance and effort, however only appraisals of intrinsic pleasantness and compatibility with internal standards were found to mediate the relationship between affiliative extraversion and warmth-affection. Intrinsic pleasantness is an assessment of how inherently pleasant an event or object is, independent of the individual’s current needs or desires. Compatibility with internal standards reflects the extent to which an event is compatible with individuals’ self-concepts, personal values and morals (Ellsworth and Scherer, 2003). Future research should therefore consider how individuals evaluate how compatible an event is with these various aspects. McConnell’s Multiple Self-Aspects Framework may be a useful model for doing so. In this model, the self consists of several self-aspects, which can comprise several constructs, such as roles, social identities, and goals (McConnell, 2011). Moreover, receiving positive feedback with regard to a particular self-aspect or attribute is expected to produce positive affect (McConnell et al., 2009; McConnell, 2011). Future research could therefore investigate whether individual differences in the content and structure of self-aspects are related to appraisals of Compatibility with Internal Standards in response to an affiliative stimulus. It is notable in this context that affiliative extraversion has previously been found to be positively associated with communal values (Trapnell and Paulhus, 2012), and so affiliative extraverts may react to affiliative scenarios more strongly because these events are appraised as being consistent with these values in particular.

Individual differences in positive activation were expected to be mediated by appraisals of intrinsic pleasantness, self-agency, effort, predictability and importance, although only importance was identified as mediating the relationship between affiliative extraversion and positive activation. Events are appraised as being important when they are pertinent to an individual’s needs or goals. The finding that importance mediates the relationship between affiliative extraversion and positive activation in response to an affiliative stimulus is consistent with previous findings that extraverts rate relationship goals as being more important than introverts (Roberts and Robins, 2000). Events that are appraised as important are also afforded additional attentional processing (Scherer, 2013), and it is therefore also noteworthy that affiliative extraversion has also been associated with an attentional bias toward affiliative stimuli (Moore et al., 2014).

More generally, a cognitive appraisal account of affective reactivity in affiliative extraversion is consistent with the view that the higher order trait of extraversion reflects individual differences in reward processing (Smillie, 2013). For example, extraverts have been found to show attentional biases toward pleasant stimuli (Paelecke et al., 2012) and greater reactivity on electrophysiological markers of reward processing in response to unpredicted reward (Cooper et al., 2014). Moreover, extraversion is also positively associated with the P300 component of event-related potentials in response to social stimuli, suggesting that these stimuli possess greater motivational significance for extraverts and are therefore allocated a greater degree of attentional processing (Fishman et al., 2011). Future research that applies these methods to the affiliative and agentic components of extraversion specifically would be useful in better understanding how cognitive processes contribute to affective reactivity in these traits.

While our findings are consistent with the hypothesis that cognitive appraisals mediate the relationships between affiliative extraversion and affect, our predictions regarding which specific appraisals would account for these relationships were largely unsupported. These predictions were based on prior research on the appraisal correlates of warmth-affection and positive activation (Ellsworth and Smith, 1988), and it may be that more accurate predictions could be made in future research by attending to the cognitive-affective processes related to affiliative and agentic extraversion. The present research will be helpful in this regard, as there is currently little data on how appraisals relate to either the affiliative or agentic extraversion.

### Limitations of the Current Research

The correlational nature of our data means that assumptions on the causal role of appraisals are tentative. Previous researchers have approached this problem by manipulating appraisals experimentally (Roseman and Evdokas, 2004), and similar manipulations of individuals’ appraisals of intrinsic pleasantness, importance and compatibility with internal standards would be helpful in testing the causal role of these appraisals in affective reactivity.

Also, the lack of previous research on how appraisals relate to agentic and affiliative extraversion prevented us from making theoretically driven predictions on which appraisals in particular would mediate the personality-affect relationships under investigation. Although we made some predictions on which appraisals might be relevant on the basis of previous research on appraisals and positive affect (Ellsworth and Smith, 1988), we largely followed an inductive approach in the current study. The exploratory nature of our analyses, coupled with a modest sample size, means that the results of this study should therefore be considered to be hypothesis generating, rather than confirmatory. It will therefore be important to undertake replication studies in future research.

This research also raises some conceptual issues that should be acknowledged. First, the present study examined affective reactivity in affiliative and agentic extraversion on measures of positive activation and warmth-affection, as these are the affective states that have been previously associated with these traits and their neurobehavioral bases (Depue and Collins, 1999; Depue and Morrone-Strupinsky, 2005). Whilst this approach is consistent with the personality literature, positive activation and warmth-affection are constructs derived from separate conceptual approaches to affect and emotion. Specifically, positive activation is a construct derived from dimensional models of affect, and represents a broad state of high-arousal pleasant affect. Warmth-affection on the other hand is a more specific affective state, and is therefore more closely aligned with models of discrete emotions. This presents a potential conceptual challenge for studies of affiliative and agentic reactivity, though it may be possible to integrate positive activation and warmth-affection within a hierarchical model of affect. This model consists of a bipolar dimension of unpleasant-pleasant affect at the top, followed by two independent dimensions of positive and negative activation, and a range of differentiated discrete affects at the bottom (Tellegen et al., 1999; Watson and Stanton, 2017).

It should also be acknowledged that this view, that appraisals cause emotions and affect, is derived from a particular set of classical appraisal models (Barrett, 2014). Barrett (2014) distinguishes between these causal models and a second set of constitutive appraisal theories however, that do not make similar claims regarding the causal role of appraisal in eliciting emotions or affect. The OCC model for example (Clore and Ortony, 2013; Ortony and Clore, 2015), holds that appraisals are simply descriptions of how individuals experience situations, and that rather than being causal antecedents of emotions, appraisals are part of the emotion itself.

## Conclusion

Affiliative extraversion was found to predict both warmth-affection and positive activation following an affiliative stimulus, and these relationships were mediated by appraisals of intrinsic pleasantness, compatibility with internal standards and importance. These findings indicate that appraisals may be one psychological mechanism that can account for affective reactivity in affiliative extraversion, although future confirmatory studies are needed to further test this hypothesis.

## Author Contributions

GI contributed to the research design, data collection, data analysis, and the interpretation of the findings. MO contributed to the design of the research and interpretation of the findings. SH contributed to the data analysis. All authors contributed to the drafting and revising of the research, and all provided final approval of the version to be published.

## Conflict of Interest Statement

The authors declare that the research was conducted in the absence of any commercial or financial relationships that could be construed as a potential conflict of interest.

## Footnotes

1 www.qualtrics.com
