# Supplementary material for: Cognitive Appraisals Mediate Affective Reactivity in Affiliative Extraversion
Source: Front Psychol. 2018 May 23;9:782. doi: 10.3389/fpsyg.2018.00782 (PMC5992422; doi:10.3389/fpsyg.2018.00782)
Supplement: Supplementary file 1 [file Table_1.DOCX]

Supplementary Material 1: Appraisal measures

Below are a series of questions about the situation that you have just imagined yourself in. Please answer these questions to indicate that you thought as you were imagining yourself in this situation.

*Intrinsic Pleasantness*

How pleasant would this family interaction be in general, regardless of your current needs, desires or feelings? / How pleasant would winning the lottery be in general, regardless of your current needs, desires or feelings?

*Importance*

How important was this family interaction to you? / To what extent was winning the lottery important to you?

To what extent did you think that this family interaction would have important consequences for you?  / To what extent did you think that winning the lottery would have important consequences for you?

Think about the things that you value in life and the goals you hope to achieve (e.g. power, wealth, family etc.).   To what extent was this family interaction relevant to any of these?  / Think about the things that you value in life and the goals you hope to achieve (e.g. power, wealth, family etc.).   To what extent was winning the lottery relevant to any of these?

*Cause: Situational-Agency*

To what extent did this family interaction occur by chance? / To what extent did you win the lottery by chance?

To what extent did this family interaction occur through natural forces outside of anyone’s control?  / To what extent did you winning the lottery due to natural forces outside of anyone's control?

To what extent did this family interaction occur randomly? / To what extent was your winning the lottery random?

*Cause: Self-Agency*

To what extent did this family interaction occur as a result of your own behaviour? / To what extent did you think that it was your own behaviour that caused the lottery win?

How responsible were you for what happened in this family interaction? / How responsible were you for winning the lottery?

How responsible were you for this family interaction occurring? / How responsible were you for the outcome of buying the lottery ticket?

*Cause: Other-Agency*

To what extent did this family interaction occur as a result of another person(s)’s behaviour? / To what extent did another person(s)’s behaviour cause the lottery win?

How responsible was another person for what happened in this family interaction? / How responsible was another person(s) for the lottery win?

How responsible was another person for this family interaction occurring? / How responsible was another person(s) for the outcome of buying the lottery ticket?

*Outcome Probability*

To what extent did you think that the outcomes of this family interaction were clearly predictable? / To what extent did you think that the outcome of buying the lottery ticket was clearly predictable?

To what extent could you have predicted what was going to happen in this family interaction? / To what extent could you have predicted what would happen when you bought the lottery ticket?

To what extent did you know how this family interaction would end? / To what extent did you know that you would win the lottery?

*Goal Conduciveness*

To what extent did you think that this family interaction would have positive consequences for you? / To what extent did winning the lottery have positive consequences for you?

To what extent would this family interaction be beneficial to you? / To what extent was winning the lottery beneficial to you?

Think about the things that you value in life, and the goals you hope to achieve (e.g. health, wealth, family etc.).  To what extent would this family interaction help you to achieve these? / Think about the things that you value in life, and the goals you hope to achieve (e.g. health, wealth, family etc.).  To what extent did winning the lottery help you to achieve these?

*Controllability*

To what extent could a person(s) (either you or another person) control what happened in this family interaction? / To what extent could a person(s) (either you or another person) control what happened after you bought the lottery ticket?

To what extent could a person(s) (either you or another person) influence the outcome of this family interaction? / To what extent could a person(s) (either you or another person) influence the outcome of buying the lottery ticket?

*Power*

To what extent did you think that *you*could influence what was happening this family interaction? / To what extent did you think that *you*could influence the outcome of buying the lottery ticket?

To what extent did you think that *you*would be able to influence or control the potential consequences of this family interaction? / To what extent did you think that *you*were able to influence or control the potential consequences of buying the lottery ticket?

*Compatibility with Internal Standards*

To what extent was this family interaction consistent with your personal beliefs, values and ideals? / To what extent was winning the lottery consistent with your personal beliefs, values and ideals?

To what extent was your behaviour in this family interaction consistent with the image you have of yourself? / To what extent was winning the lottery consistent with the image you have of yourself?

To what extent was your behaviour in this situation consistent with your morals? / To what extent was winning the lottery consistent with your morals?

*Effort*

How much effort (mental or physical) did you feel you had to expend during this family interaction? / How much effort (mental or physical) did you feel you had to expend during this situation?

Think about what you wanted in this family interaction – to what extent did you feel there were obstacles standing in the path between you and getting what you wanted? / Think about what you wanted in this situation – to what extent did you feel there were obstacles standing in the path between you and getting what you wanted?

To what extent did you feel that you had to exert yourself (mentally or physically) to get what you wanted in this family interaction? / To what extent did you feel that you had to exert yourself (mentally or physically) to get what you wanted from this situation?

*Fairness*

To what extent did do think that you deserved something good to happen in this family interaction? / To what extent did do think that you deserved something good to happen when you bought the lottery ticket?

To what extent do you think what happened to you in this family interaction was fair? / To what extent do you think winning the lottery was fair?

To what extent do you think that you deserved what happened in this family interaction? / To what extent do you think that you deserved to win the lottery?
